# Extra Virgin Olive Oil and *Nigella sativa* Oil Produced in Central Italy: A Comparison of the Nutrigenomic Effects of Two Mediterranean Oils in a Low-Grade Inflammation Model

**DOI:** 10.3390/antiox9010020

**Published:** 2019-12-24

**Authors:** Laura Bordoni, Donatella Fedeli, Dennis Fiorini, Rosita Gabbianelli

**Affiliations:** 1 School of Pharmacy, Unit of Molecular Biology, University of Camerino, Via Gentile III da Varano, 62032 Camerino, MC, Italy; laura.bordoni@unicam.it (L.B.); donatella.fedeli@unicam.it (D.F.); 2 School of Science and Technology, Chemistry Division, University of Camerino, Via Sant’Agostino, 62032 Camerino, MC, Italy; dennis.fiorini@unicam.it

**Keywords:** nutrigenomics, epigenetics, low-grade inflammation, extra virgin olive oil, *Nigella sativa*

## Abstract

Extra virgin olive (EVO) oil and *Nigella sativa* (NG) oil are two well-known Mediterranean foods whose consumption has been associated with beneficial effects on human health. This study investigates the nutrigenomic properties of two high quality EVO and NG oils in an in vitro model of low-grade inflammation of human macrophages (THP-1 cells). The aim was to assess whether these healthy foods could modulate inflammation through antioxidant and epigenetic mechanisms. When THP-1 cells were co-exposed to both lipopolysaccharides (LPS)-induced inflammation and oils, both EVO and NG oils displayed anti-inflammatory activity. Both oils were able to restore normal expression levels of *DNMT*3*A* and *HDAC1* (but not *DNMT*3*B*), which were altered under inflammatory conditions. Moreover, EVO oil was able to prevent the increase in *TET2* expression and reduce global DNA methylation that were measured in inflamed cells. Due to its antioxidant properties, EVO oil was particularly efficient in restoring normal levels of membrane fluidity, which, on the contrary, were reduced in the presence of inflammation. In conclusion, these data support the hypothesis that these Mediterranean oils could play a major role in the modulation of low-grade inflammation and metabolic syndrome prevention. However, NS oil seems to be more efficient in the control of proinflammatory cytokines, whereas EVO oil better helps to counteract redox imbalance. Further studies that elucidate the nutrigenomic properties of local produce might help to promote regional the production and consumption of high-quality food, which could also help the population to maintain and promote health.

## 1. Introduction

Inflammation is an essential component of innate immunity, but it can act as both a “friend and foe”. It plays a major role in immunosurveillance and host defense [1], but a chronic low-grade inflammatory state also represents the start of a wide range of chronic and multifactorial pathologies (i.e., metabolic syndrome, type 2 diabetes mellitus, cardiovascular disease [2,3], non-alcoholic fatty liver disease and neuroinflammation [4]). Indeed, the control of low-grade inflammation is an important step in the prevention of some of the most prevalent and severe human pathologies.

It has been demonstrated that the resolution of inflammation is an active process involving cytokines and other anti-inflammatory mediators, particularly lipids, rather than simply being the switching off of pro-inflammatory pathways [5,6]. Numerous dietary components have the potential to modulate the predisposition to chronic low-grade inflammatory conditions, and consequently, they might play a role in the prevention and treatment of numerous complex diseases [7,8]. These food components (including, for instance, omega-3 fatty acids, plant flavonoids, vitamins, pre and probiotics) act through different kinds of mechanisms ranging from antioxidant effects to gene expression (nutrigenomic) and cell-signaling modulation, to promoting the function of the gut barrier and anti-inflammatory responses [7]. In particular, gut permeability to bacterial lipopolysaccharides (LPS), a potent inflammatory stimulant, appears to be an important trigger for low-grade systemic inflammation; through the interaction with mononuclear cells, LPS may be an important trigger in the development of inflammation and metabolic diseases [9]. Moreover, among various pathways, epigenetics has also been demonstrated to play a role in inflammation [10]. Indeed, growing evidence associates epigenetic marks (DNA methylation, histone modifications, non-coding RNA and chromatin structure) with chronic inflammatory diseases [11,12]. In this context, the potential role of many bioactive nutrients in regulating human health (also through effects on epigenetics) has become evident, and nutri(epi)genomics has emerged as a new and promising field in current nutritional research [13,14].

Extra virgin olive (EVO) oil and *Nigella sativa* (NG) oil are two Mediterranean foods whose consumption has been demonstrated to have beneficial effects on human health [15,16,17,18,19,20,21,22,23,24,25].

EVO oil (EVOO) has been indexed as one of the main dietary constituents responsible for the health benefits attributed to the Mediterranean diet. The beneficial effects have been attributed to the high monounsaturated fatty acids content, in particular to oleic acid, and also to a combination of several phytochemicals (more than 200 minor components, i.e., tyrosol, hydroxytyrosol, oleocanthal, tocopherols, β-carotene, lutein, squalene, vanillic acid, gallic acid, pinoresinol, luteolin and many others) that have different biological activities (antioxidant, anti-inflammatory, antimicrobial, antiviral, anti-atherogenic, anti-thrombotic, anti-mutagenic and hypoglycemic) [26,27]. These bioactive compounds are probably responsible for EVOO oxidative stability, sensorial attributes, antioxidant, anti-inflammatory and immunomodulatory activity [23]. Despite promising evidence on the health effects of EVOO, few data on the epigenetic properties of EVOO are currently available [28,29], with most studies focused on miRNA or histone modifications [24]. Previous studies concerning the chemical properties (fatty acid composition and α-tocopherol content), antioxidant activity and sensory characterization of several Mediterranean EVO oils have highlighted the significant antioxidant properties of EVO oil coming from the Raggia cultivar of the Marche region in Central Italy [30]. These preliminary data indicate that this specific EVO oil is a particularly healthy food that is potentially able to exert beneficial effects against inflammation.

Another interesting Mediterranean oil is NG oil. Many pharmacological actions such as antioxidant, antimicrobial, anti-inflammatory, immunomodulator, analgesic, anti-arthritic, anti-diabetic, anti-asthmatic and anti-cancer have been attributed to *Nigella sativa* [15,18,19,31] and in particular to thymoquinone, which is one of the most bioactive components of this oil [15,18,20,32,33,34]. Due to its properties, NG is regarded in the Middle East as part of an overall holistic approach to health and is thus incorporated into diets and everyday lifestyles [18]. A specific variety of NG oil produced in the Marche region in Italy has been demonstrated to have high quality and beneficial properties against inflammation in pre-adipocytes [35]. The thymoquinone concentration, antioxidant properties, fatty acid composition and tocopherol quantification of this NG oil have been characterized [31,35].

Thus, the present study aims to investigate the nutrigenomic properties of these high-quality EVO and NG oils in an in vitro model of low-grade inflammation of human macrophages, in order to potentially elucidate molecular pathways triggered by these healthy foods.

## 2. Materials and Methods

### 2.1. Reagents and Cells Culture

All reagents were of analytic grade and purchased from Sigma Chemical Co. St. Louis (St. Louis, MO, USA) if not otherwise stated. Both oils were produced in the Marche region, Italy; the EVO oil belongs to the Raggia cultivar and the *Nigella sativa* oil was produced by the Tre Ponti Snr company, Polverigi (AN), Italy. *Nigella sativa* seeds were stored at a controlled temperature (14 °C) in a dark room. Oil was extracted by cold pressing seeds using a squeezing machine (Vero Energia Italia S.r.l., Ravenna, Italy), and filtered after 10 days to remove solid residues. The human monocytic cell line (THP-1) was purchased from the Istituto Profilattico Sperimentale della Lombardia e dell’Emilia Romagna Bruno Ubertini (Brescia, Italy).

### 2.2. Cytotoxicity Assay

Cytotoxicity in THP-1 cells after 24, 48 and 72 h of incubation with EVO and NG oil samples was tested by the 3-(4,5-dimethylthiazol-2-yl)-2,5-diphenyltetrazolium bromide (MTT) assay (Fisher Scientific, Italia, Italy). Cells were plated in a 96-well plate at a seeding density of 1 × 10^4^ cells/well. After 24 h, cells were treated with NG oil as described in our previous paper [35]. Briefly, the concentrations of NG oil (based on the thymoquinone concentration: A: 100 μM, B: 50 μM, C: 10 μM, D: 5 μM, E: 1 μM, F: 0.5 μM) that were previously tested in SGBS pre-adipocytes were also tested in the THP-1 cell line. The cytotoxicity of the same volumes of EVO oil (A,B,C,D,E,F) was tested in the same model. At the end of the treatment period (24, 48 or 72 h), 50 μL of MTT (5 mg/mL in phosphate buffered saline) solution were added to each well. After 4 h of incubation at 37 °C, MTT was discarded, the formazan crystals were dissolved in 100 μL of dimethylsulfoxide (DMSO) and absorbance at 570 nm was measured after 10 min using an ELISA reader (Fluostar Omega, BMG Labtech, Ortenberg, Germany).

### 2.3. In Vitro Model of Low-Grade Inflammation

**Figure 1:**
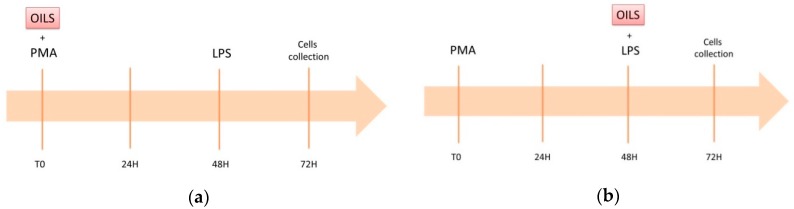
Graphical representation of the two different experimental settings: (**a**) SET1, exposure to oils (NG or EVO) occurs from the beginning of the differentiation; (**b**) SET2, exposure to oils (NG or EVO) occurs at the time of inflammation induction with lipopolysaccharides (LPS).

THP-1 cells were cultured in RPMI complete medium containing 2 mM L-glutamine, 10% fetal bovine serum (FBS) and 50 μM 2-mercaptethanol. The cells were maintained in a humidified atmosphere containing 5% CO_2_ at 37 °C in an incubator. A low-grade inflammation model was established as previously described by Park and collaborators [36]. Briefly, THP-1 cells were treated for 48 h with 5 ng/mL of Phorbol 12-myristate 13-acetate (PMA), which is the minimal concentration required to induce stable differentiation without undesirable gene upregulation. Then, cells were treated for 24 h with 20 ng/mL of lipopolysaccharide (LPS), as it has been demonstrated that macrophages differentiated at 5 ng/mL responded well to such a weak secondary stimuli [36]. This model was previously suggested as a suitable tool to study the inflammation-modulating effects of food-derived compounds [37]. Activated macrophages were exposed to the oils under two different experimental regimens: (a) pre-treatment with PMA for 72 h; and (b) co-treatment with LPS for 24 h (Figure 1). The oils were dissolved in DMSO.

### 2.4. Gene Expression Assessment

RNA was extracted from frozen cell pellets by using the Aurum Total RNA Fatty and Fibrous Tissue Kit (Bio-Rad Laboratories, Hercules, CA, USA) and then retro-transcribed using iScript cDNA Synthesis Kit (Bio-Rad Laboratories, USA) following the manufacturer’s instructions. RT-PCR was performed through a SYBER green-based assay using 50 ng of cDNA for each reaction and Biorad CFX96 (Bio-Rad Laboratories, USA) as the detector system. ΔΔCT method normalizing for HPRT was performed in order to calculate relative fold expression changes. Primers were purchased from Metabion (Metabion International AG, Planegg, Germany) and the sequences are available in the Table S1.

### 2.5. Global DNA Methylation and Hydroxymethylation

DNA was isolated from frozen pellets by using DNAzol (Thermo Fisher Scientific, Waltham, MA, USA) according to the protocol provided by the company and quantified by using the NanoDrop (Thermo Fisher Scientific, USA). Global DNA methylation and hydroxymethylation were determined by ELISA colorimetric assays (Methylated DNA Quantification Kit, ab117128; Hydroxymethylated DNA Quantification Kit, ab117130A; bcam, Cambridge, UK;) according to the manufacturer’s instructions.

### 2.6. MtDNA Copy Number Evaluation

MtDNA copy number was assessed as previously described [38] by a qPCR method using primers specific for mtDNA (mtpair21 fw-AATCCAAGCCTACGTTTTCACA; mtpair21 rv-AGTATGAGGAGCGTTATGGAGT) and normalizing for the amount of genomic DNA used in each reaction by using primers specific for gDNA (18s fw-GCAATTATTCCCCATGAACG; 18s-rv GGGACTTAATCAACGCAAGC). The qRT reaction was performed using Takara TB Green™ Premix Ex Taq™ II (Takara) and by using CFX-96 (Biorad).

### 2.7. Reduced Glutathione to Oxidized Glutathione (GSH/GSSG) Ratio Measurement

The measurement of GSH and GSSG was performed by a fluorometric assay using O-phthalaldehyde (OPT) as a fluorescent reagent, as previously described by Singh and collaborators [39]. OPT has the ability to bind GSH at pH 8 and GSSG at pH 12. N-ethylmaleimide is used to prevent the autoxidation of GSH to GSSG during the determination of GSSG in the sample. Briefly, about 1 × 10^6^ THP-1 cells were suspended in KPE buffer (potassium phosphate buffer + EDTA 0.1 M, pH 8) and the protein concentration was determined using the Pierce BCA Protein Assay Kit (Thermo Scientific). A 10 µg protein sample was precipitated with a solution of 50% trichloroacetic acid (TCA) in a ratio of 4:1, vortexed and kept on ice for 10 min. The protein sample with TCA was then centrifuged at 9100× *g* for 10 min at 4 °C and the supernatant transferred into a fresh 1.5 mL centrifuge tube. For GSH estimation, 10 µL supernatant was mixed with an equal volume of OPT (1 mg/mL) and 180 µL KPE buffer (pH 8) in a black 96-well plate while for GSSG estimation, 50 µL of the supernatant was mixed with 0.5 µL N-ethylmaleimide (stock concentration: 4 M) and incubated for 30 min at room temperature to inhibit GSH. Ten µL of this sample was treated with 10 µL OPT and 180 µL 0.1 N NaOH (pH 12) in a black 96-well plate and incubated at room temperature for 10 min. Finally, the fluorescence at emission wavelength of 420 nm was determined using an excitation wavelength of 355 nm in a microplate reader. The concentration of GSH and GSSG were obtained by comparison with a standard curve using both as standards.

### 2.8. Membrane Fluidity

Membrane fluidity on THP-1 cells, treated as previously described, was assessed by a fluorescence assay using 6-lauroyl-2-dimethylaminonaphthalene (Laurdan) (Molecular Probes (Eugene, OR, USA) as a probe to detect the lateral mobility and polarity of the membrane environment, and thus water penetration into the hydrophobic part of the bilayer. THP-1 cell samples were normalized at a final protein concentration of 25 µg/mL. The assay was done in 1 mL of sodium phosphate buffer (2.5 mM, pH 7.4), containing 25 µg of protein and 1 µM of Laurdan. Fluorescence was determined on a Hitachi fluorimeter using an excitation wavelength of 340 nm and emission at 440 and 490 nm wavelengths. The generalized polarization of Laurdan (GP340) was calculated according to the Parasassi equation [40]:

GP340 = (Ib − Ir)/(Ib + Ir)(1)

where Ib and Ir are the intensities at the blue (440 nm) and red (490 nm) edges of the emission spectrum and correspond to the fluorescence emission maximum in the gel and liquid-crystalline phases of the bilayer, respectively [41].

### 2.9. Statistical Analysis

Data were analyzed by the Statistical Package for Social Science (SSPS, IBM, USA). Statistically significant differences were evaluated using unpaired *t*-test or one-way ANOVA with Bonferroni correction. The level of statistical significance was defined by a two-tailed *p* value <0.05 throughout the study.

## 3. Results

### 3.1. NG but Not EVO Oil Shows Cytotoxic Properties

**Figure 2:**
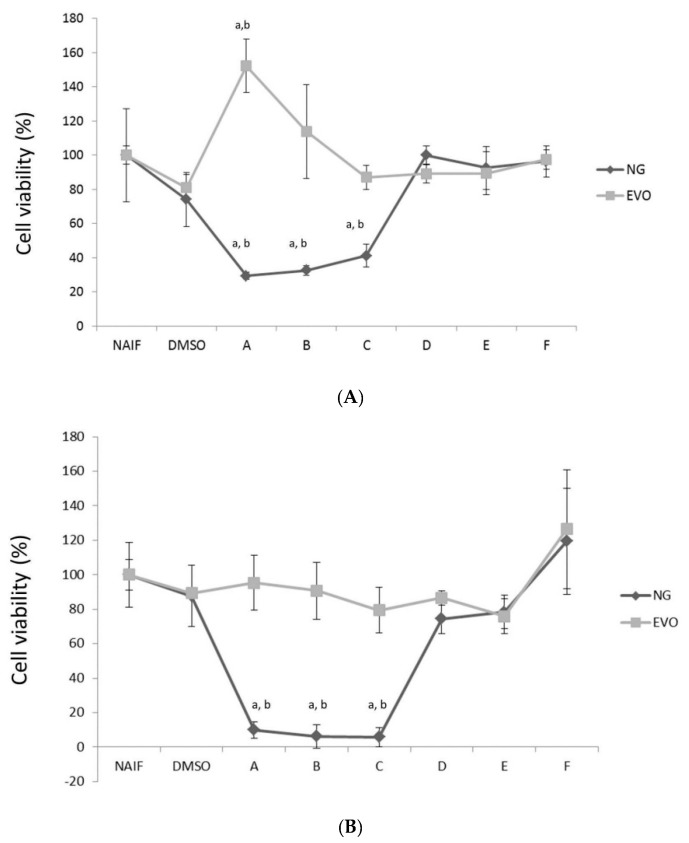

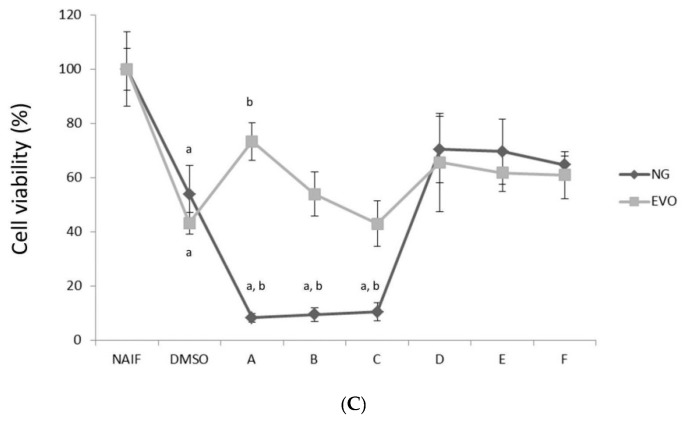
Cytotoxicity of the oils at different times, 24 h (**A**), 48 h (**B**), 72 h (**C**) and concentrations. The x axis shows the different concentrations of thymoquinone (A: 100 μM, B: 50 μM, C: 10 μM, D: 5 μM, E: 1 μM, F: 0.5 μM) contained in the *Nigella sativa* (NG) oil. Equivalent volumes of extra virgin olive (EVO) oil were tested with the same conditions. Statistically significant differences are indexed as follows: a = *p* < 0.01 vs. NAIF, b = *p* < 0.01 vs. DMSO.

No cytotoxic effects were observed for EVO oil at all concentrations or times (Figure 2). A mild increase in cell viability was observed at 24 h at the maximum concentration tested (A vs. DMSO, *p* < 0.001) (Figure 2A). On the other hand, the MTT assay on THP-1 cells confirmed that a thymoquinone concentration >5 μM reduces cell viability at 24, 48, 72 h (DMSO vs. A, B, C; *p* < 0.001). In detail, the IC_50_ for NG oil was: 35.3 μM after 24 h, 8.3 μM after 48 h and 6.4 μM after 72 h. This is consistent with what we observed in our previous study [35], where we measured a cytotoxic effect for thymoquinone concentrations ≥5 μM on SGBS cells. Thus, the volume of NG oil that provided a final content of 1 μM of thymoquinone (i.e., 0.228 μL/mL) was chosen as a suitable to observe the protective effects while avoiding any potential cytotoxicity on the model. 

### 3.2. Both NG and EVO Oils Have Anti-Inflammatory Effects after 24 h of Exposure

**Figure 3:**
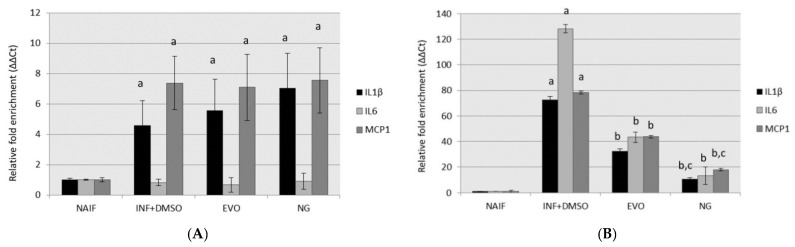
Gene expression of inflammatory genes after the treatment for the two experimental conditions: (**A**) SET1; (**B**) SET2. Statistically significant differences are indexed as follows: a = vs. NAIF; b = vs. INF + DMSO; c = vs. EVO.

Analysis of the expression of inflammatory genes (IL-1β, IL-6, MCP1) revealed that no observable effects could be measured when the treatment with oils occurred at the time of differentiation (SET1) (Figure 3A). On the other hand, both EVO (*p* < 0.01) and NG (*p* < 0.01) oils displayed anti-inflammatory effects after 24 h from exposure, when the cells were treated with oils at the time of inflammation induction with LPS (SET2) (Figure 3B). Under the same condition, NG oil showed a stronger effect than EVOO, especially in the suppression of IL-1β (*p* < 0.05) and MCP1 (*p* < 0.05) genes. 

### 3.3. Modulation of DNMTs and HDACs

**Figure 4:**
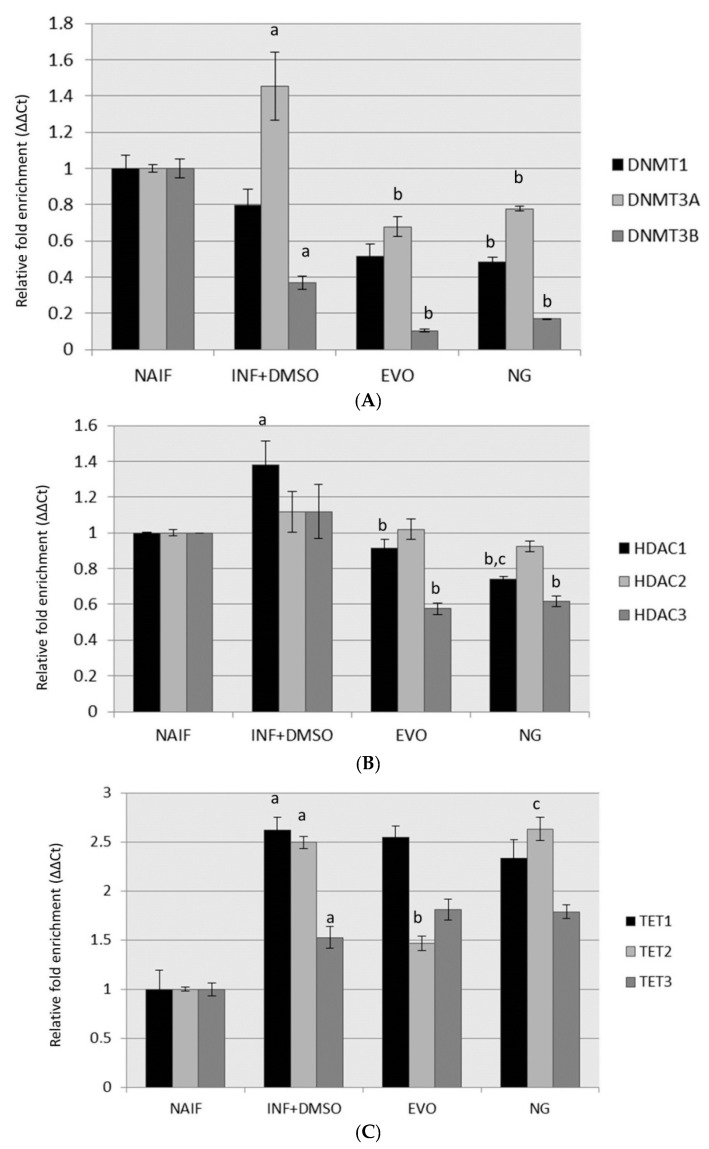
Gene expression assessment after the treatments in experimental setting 2 (SET2) for (**A**) genes regulating DNA methylation; (**B**) genes regulating histone acetylation; (**C**) genes associated with DNA demethylation. Statistically significant differences are indexed as follows: a = vs. NAIF; b = vs. INF + DMSO; c = vs. EVO.

No changes in *DNMTs*, *HDACs* and TET proteins were detected in cells pre-treated with oils (SET1) after 72 h (data not shown). On the other hand, gene expression analysis revealed increased levels of *DNMT*3A (*p* < 0.01) and *HDAC*1 (*p* < 0.05) and reduced levels of *DNMT*3B (*p* < 0.05) under inflammatory conditions after 24 h from LPS exposure compared to controls. The expression of *DNMT*3A and *HDAC*1 was restored to normal levels after treatment with both oils (for both genes: naif vs. NG, *p* > 0.05; naif vs. EVO, *p* > 0.05). On the other hand, the levels of *DNMT*3B remained lower than the controls after both NG (*p* < 0.01) and EVO (*p* < 0.01) oil treatments. A reduction in *HDAC*3 levels with respect to both naif and inflamed cells was observed after treatment with both oils (*p* < 0.05). Moreover, when the cells were co-treated with the oils (SET2), inflammation stimulated TET protein expression compared to control (*TET1*, *p* = 0.02; *TET2*, *p* = 0.03; *TET3*, *p* = 0.01); the only preventive effect observed was exerted by EVO oil, whose treatment prevented the increase in *TET2*, restoring it to normal levels with respect to controls (*TET2*, *p* > 0.05). The upregulation of *TET2* remained as high as in the inflamed cells in all of the other treatments. The results are displayed in Figure 4. *DNMT*1 expression was not altered by inflammation, but it was reduced by the exposure to NG oil (naif vs. NG, *p* < 0.05).

### 3.4. Global DNA Methylation and Hydroxymethylation Alteration

**Figure 5:**
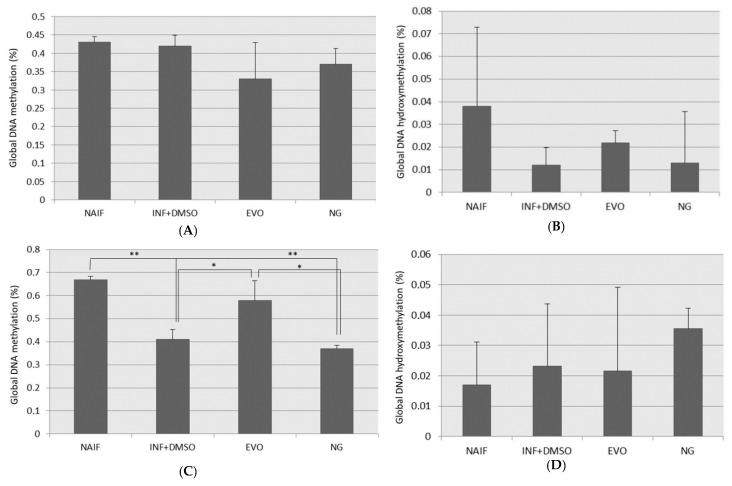
Global DNA methylation and hydroxymethylation levels measured under the different conditions for the two experimental settings: (**A**) DNA methylation for SET1; (**B**) DNA hydroxymethylation for SET1; (**C**) DNA methylation for SET2; (**D**) DNA hydroxymethylation for SET2; *: *p* < 0.05; **: *p* < 0.01.

No significant changes in DNA methylation (Figure 5A) and hydroxymethylation (Figure 5B) could be observed in the samples pretreated with oils (SET1). On the other hand, a mild reduction in DNA methylation was measured under the inflamed condition after 24 h from LPS exposure (*p* < 0.01) (Figure 5C); this evidence is consistent with the increased expression of all the TET proteins described in the previous paragraph. Moreover, the reduction in DNA methylation was counterbalanced by EVO oil treatment (EVO vs. INF, *p* = 0.025), but not NG oil (NG vs. INF, *p* > 0.05). No significant changes in global levels of DNA hydroxymethylation were observed in this set of samples (Figure 5D).

### 3.5. mtDNA Copy Number

**Figure 6:**
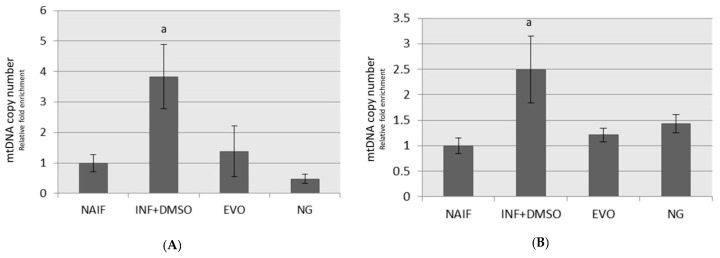
MtDNA copy number assessment after treatment under the two experimental conditions: (**A**) SET1; (**B**) SET2. a: *p* < 0.05.

**Figure 7:**
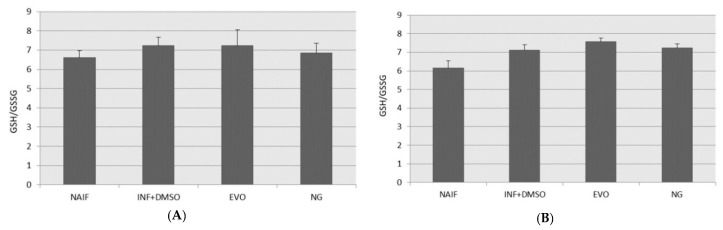
Reduced glutathione to oxidized glutathione (GSH/GSSG) ratio after treatment under the two experimental conditions: (**A**) SET1; (**B**) SET2.

Higher mtDNA copy numbers were measured in inflamed monocytes than in controls after 72 h (*p* < 0.01) and 24 h (*p* < 0.05) from the exposure (Figure 6A,B). The mtDNA copy number was restored to levels similar to the controls after treatment with EVO and NG oils under both the experimental regimens (Figure 7).

### 3.6. GSH/GSSG Levels

GSH plays a key role in redox control at the level of the cytosol and mitochondria; GSH works non-enzymatically with several kinds of oxidants, and its concentration decreases during acute inflammation with the formation of GSSG. In the present study, no significant alterations in the GSH/GSSG ratio could be measured under these experimental conditions among all the different treatments (Figure 7A,B).

### 3.7. Cell Membrane Fluidity

**Figure 8:**
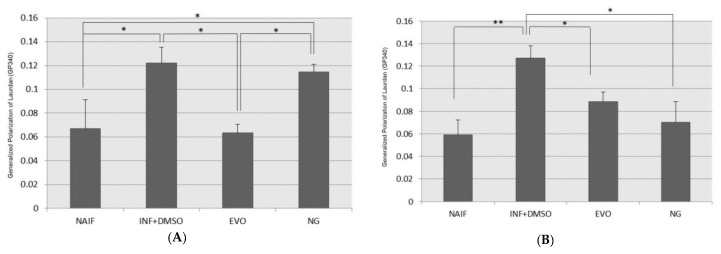
Assessment of membrane fluidity after treatment under the two experimental conditions: (**A**) SET1; (**B**) SET2. *: *p* < 0.05; **: *p* < 0.01.

Plasma membrane fluidity can be modified by lipid composition and endogenous or exogenous stimuli such as oxidants or inflammation; the increased number of bonds between lipids at different depths can reduce their mobility leading to a decrease in the fluid state of the membrane. A decrease in membrane fluidity was measured in inflamed cells, under both experimental designs (Figure 8A,B). EVO oil restored normal levels of membrane fluidity both after 72 h (SET1) and 24 h (SET2). NG oil was able to restore normal levels of membrane fluidity only after 24 h, when the exposure was in co-treatment with LPS (SET2).

## 4. Discussion

EVO and NG oils are interesting Mediterranean products to which health properties have been attributed [21,34]. While EVOO is a typical Italian food, *Nigella sativa* is rarely found in Italy. Interestingly, this specific NG oil produced in the Marche region displayed remarkable anti-inflammatory properties and high amounts of thymoquinone compared to those from other Mediterranean countries [35]. This study investigated the nutrigenomic properties of two different oils, both produced in Central Italy, in order to investigate if they were able to modulate gene expression and epigenetic pathways under inflammatory conditions. The experimental design included a particularly interesting model where a low-grade inflammation was set up; therefore, the effects of food compounds on health are investigated in a way that is more similar to normal physiological conditions while avoiding acute inflammatory stimuli (against which food could only marginally have an effect). The results suggest that both these two Mediterranean oils (probably because of the activity of different bioactive compounds) can counteract the low-grade inflammation status, which is one of the major contributors to non-communicable diseases affecting western countries. Moreover, both EVO and NG oils modulated epigenetic pathways, therefore exerting anti-inflammatory effects through this property. Few studies on EVOO and epigenetic modulation have been conducted. Recent papers have reported that EVOO supplementation can modulate DNA methylation in vivo [42], and downregulate *HDAC*2 and *HDAC*3; this second effect in particular might be exerted, as newly hypothesized, by a specific compound: oleuropein [43]. On the other hand, thymoquinone is probably the main compound responsible for modulation of gene expression observed in cells treated with NG oil. Indeed, although *DNMT*1 expression was not associated with inflammation in our model, a moderate downregulation is exerted by NG oil in THP-1 cells. This is consistent with previous investigations suggesting that thymoquinone could reverse DNA hypermethylation in cancer cells [44,45]. Another recent study demonstrated that thymoquinone can decrease the expression of some important epigenetic proteins like *DNMT*1,3A,3B and *HDAC*1 in Jurkat cells and breast cancer cells [46]. In fact, it has been recently speculated that because of its structure, thymoquinone could act as both a demethylating and methylating agent [45]. Moreover, our data showed that the low-grade inflammation status increases TET protein expression; in the experimental condition tested in this study, only EVOO was able to counterbalance *TET2* increase and to maintain its expression levels at the levels of controls. On the other hand, NS oil (probably due to its thymoquinone content) is more active than EVOO in the regulation of proinflammatory cytokine release, but less efficient in counteracting unbalanced redox control. Indeed, the results suggest that only EVOO can protect against the changes in membrane fluidity induced by low-grade inflammation. We can speculate that this property could result in more efficient control of plasma membrane protein activity, and hence it could also impact cellular and nuclear responses. EVO and NS oils have previously been evaluated for their chemical composition and antioxidants properties [30,47] and significant differences have been identified; total antioxidant activity, DPPH radical-scavenging activity, superoxide scavenging activity and catalase-like activity were +77%, +62%, +90%, +98% and +91% higher in EVOO than in NS oil [30]. The higher antioxidant properties of EVOO can be explained by the much higher concentration of the most active tocopherol (α-tocopherol), found in EVOO at 284 mg kg^−1^ and in NS oil at 27 mg kg^−1^ [31,47] and to the presence many other strong antioxidants in EVOO such as tyrosol, hydroxytyrosol and secoiridoid derivatives found in relatively high concentration and absent in NS oil. In particular, tyrosol, hydroxytyrosol and the main secoiridoid derivatives (the dialdehydic form of decarboxymethyl elenolic acid linked to hydroxytyrosol or to tyrosol, an isomer of the oleuropein aglycon, and ligstroside aglycon), were found in the EVOO under investigation at concentrations of 6.7, 9.8 and 583.6 mg kg^−1^, respectively [30,47]. These substances are recognized as being those mainly responsible for the antioxidant activity of EVOO. Their effect on the protection of blood lipids from oxidative stress has been demonstrated in several studies, thus allowing the acknowledgement by the European Food Safety Authority of a health claim that can apply to olive oils containing a minimum of 250 mg kg^−1^ of the mentioned phenolic substances [48]. On the other hand, NS oil is characterized by its high content of thymoquinone (7.2 mg/mL), which has anti-inflammatory properties [35]. 

Thus, the compositional differences found between the two oils can help to explain their different properties, and hence, could be responsible for the biological effects investigated in the low-grade inflammation model.

Finally, the nutrigenomic properties of these oils are also reflected at the level of mitochondrial genome regulation. Since mtDNA content has been inversely associated with inflammatory markers [49,50], the increased copy number observed in our inflamed samples is probably an attempt by the cell to counterbalance the inflammatory process; this phenomenon does not occur in cells treated with both oils.

## 5. Conclusions

In accord with previous evidence measured in human adipocytes [35], these data support the hypothesis that these Mediterranean products could play a major role in the modulation of low-grade inflammation and metabolic syndrome prevention. However, these two specific oils displayed interesting healthy properties; NS oil seems to be more efficient in the control of proinflammatory cytokines, whereas EVOO better helps to counteract redox imbalance. Therefore, they both represent interesting possibilities to prevent and control low-grade inflammation thus counteracting the onset of complex diseases by dietary and lifestyle changes. This evidence could open the way to further studies aimed to corroborate the epigenetic-mediated anti-inflammatory actions by investigating the effect of these Mediterranean oils in in vivo models of inflammation, in order to identify effective dosage and timing of administration that could be translated into clinical practice. Identifying the nutrigenomic properties of local products helps to promote regional production of high-quality foods, whose consumption could help the population to maintain and promote health.

## Supplementary Materials

The following are available online at https://www.mdpi.com/2076-3921/9/1/20/s1, Table S1: Sequences of primers used for gene expression analysis.
