# Supplementary material for: Extra Virgin Olive Oil and Nigella sativa Oil Produced in Central Italy: A Comparison of the Nutrigenomic Effects of Two Mediterranean Oils in a Low-Grade Inflammation Model
Source: Antioxidants (Basel). 2019 Dec 24;9(1):20. doi: 10.3390/antiox9010020 (PMC7022781; doi:10.3390/antiox9010020)
Supplement: Supplementary file 1 [file antioxidants-09-00020-s001.pdf]

Gene	Forward Primer	Reverse Primer
**IL1b**	TGAAAGATGATAAGCCCACTCTACA	AGACTCAAATTCCAGCTTGTTATTG
**IL6**	TACCCCCAGGAGAAGATTCC	TTTTCTGCCAGTGCCTCTTT
**MCP1**	TCCCAAAGAAGCTGTGATCTTCAAGACC	AGTGAGTGTTCAAGTCTTCGGAGTTTGG
**TET1**	CAGAACCTAAACCACCCGTG	TGCTTCGTAGCGCCATTGTAA
**TET2**	GATAGAACCAACCATGTTGAGGG	TGGAGCTTTGTAGCCAGAGGT
**TET3**	TCCAGCAACTCCTAGAACTGAG	AGGCCGCTTGAATACTGACTG
**HDAC1**	CATCTCCTCAGCATTGGCTT	CGAATCCGCATGACTCATAA
**HDAC2**	ATGAGGCTTCATGGGATGAC	ATGGCGTACAGTCAAGGAGG
**HDAC3**	CTGTGTAACGCGAGCAGAAC	GCAAGGCTTCACCAAGAGTC
**HPRT**	GAGATGGGAGGCCATCACATTGTAGCCCTC	CTCCACCAATTACTTTTATGTCCCCTGTTGACTGGTC
**DNMT1**	PrimePCR™ SYBR® Green Assay: DNMT1, Human; assay ID: qhsacid0021977	
**DNMT3A**	PrimePCR™ SYBR® Green Assay: DNMT3A, Human; assay ID:qhsaced0042577	
**DNMT3B**	PrimePCR™ SYBR® Green Assay: DNMT3B, Human; assay ID: qhsacid0010863	
